# A Population-Based Study of COVID-19 Infection Among Childhood Cancer Survivors

**DOI:** 10.3389/fmed.2021.718316

**Published:** 2021-09-07

**Authors:** Mohammad Agha, Felicia Leung, Rahim Moineddin, Nicole M. Bradley, Paul J. Gibson, David C. Hodgson

**Affiliations:** ^1^Pediatric Oncology Group of Ontario, Toronto, ON, Canada; ^2^ICES, Toronto, ON, Canada; ^3^Department of Family and Community Medicine, University of Toronto, Toronto, ON, Canada; ^4^Department of Pediatrics, McMaster University, Hamilton, ON, Canada; ^5^Department of Radiation Oncology, University of Toronto, Toronto, ON, Canada

**Keywords:** childhood cancer, cancer survivors, COVID-19, survivorship, infection

## Abstract

Childhood cancer survivors are known to be at risk of chronic co-morbidities, although their risk of COVID-19 infection remains uncertain. Understanding the risk of COVID-19 in this population is necessary to counsel survivors and inform potential mitigation strategies. The objective of this study was to determine whether the rates of COVID-19 infection differed between childhood cancer survivors and the general population. Administrative health care data from a population-based registry of children and adolescents diagnosed with cancer in Ontario, Canada, were linked with a universal health insurance registry and a repository of laboratory data. Rates of COVID-19 testing, test positivity and infection between March 1, 2020 and March 31, 2021 among childhood cancer survivors (*n* = 10 242) were compared to matched controls from the general population (*n* = 49 068). Compared to the general population, childhood cancer survivors were more likely to have COVID-19 testing (35.9% [95% CI, 34.5–37.4%] vs. 32.0% [95% CI, 31.4–32.6%]), but had a lower likelihood of positive COVID-19 result among those tested (4.3% [95% CI, 3.6–4.9%] vs. 5.5% [95% CI, 5.1–5.8%]) and a similar rate of infection among all subjects at risk (1.5% [95% CI, 1.3–1.8%] vs. 1.7% [95% CI, 1.6–1.9%]). These findings can inform counseling of survivors and clinician recommendations for this population.

## Introduction

Consensus recommendations for childhood cancer survivors (CCS) have been developed to provide guidance to CCS and their clinicians regarding COVID-19 infection, although they are based on very limited data (1). To address this gap, we compared the COVID-19 testing, positivity and infection rates among a population-based cohort of CCS to the rates seen in a matched cohort from the general population in Ontario, Canada.

## Methods

Individuals diagnosed with cancer at age ≤ 18 years between 1985 and 2016 who survived ≥5 years and were alive on January 1, 2020 were identified from the Pediatric Oncology Group of Ontario Networked Information System (POGONIS). POGONIS is an active, population-based registry of children diagnosed with cancer at Ontario's five specialized childhood cancer programs that captures standardized demographic, diagnosis, outcome and treatment information on all registered cases and has been detailed previously (2, 3).

Population controls were identified from Ontario's Registered Persons Database, which is a population-based registry capturing the majority of Ontario's 14.5 million population and consisting of basic demographic information about individuals with public health care eligibility in Ontario. A maximum of five controls were randomly selected from those individually matched to each CCS based on year and month of birth, sex, and geographic area of residence (first three digits of postal code).

COVID-19 testing data were obtained from the Ontario Laboratory Information System (OLIS), an Ontario-wide repository of lab tests and results. Starting April 7, 2020, complete daily feeds of all COVID-19 test orders have been recorded in OLIS, extracted from lab orders with test requests or Logical Observation Identifiers Names and Codes indicative of viral or respiratory virus testing specific to COVID-19. This dataset represents the complete COVID-19 testing result status for each tested individual in Ontario on each day. CCS and controls were linked to OLIS using a unique, numeric identifier to obtain COVID-19 testing dates and results between March 1, 2020 and March 31, 2021.

### Statistical Analysis

We calculated the proportion of individuals undergoing COVID-19 testing at least once (testing rate), the proportion of tested individuals with at least one positive COVID-19 test (positivity rate) and the proportion of all subjects with at least one positive test (infection rate) among CCS and matched controls. These rates were calculated for strata of current age, time since cancer diagnosis and initial cancer diagnosis. In an analysis planned *a priori*, we evaluated these outcomes among survivors who had undergone an allogeneic bone marrow transplant (ABMT).

In multivariable analyses, we used general estimating equations with robust variance estimators to compare testing rates, positivity rates and infection rates between survivors and controls while adjusting for matching and three-month calendar periods of testing to account for variation in the positivity rates over time. This analysis included (where applicable) multiple tests per individual. Two-sided *P* < 0.01 were considered statistically significant to account for multiple testing. Analyses were conducted using SAS Enterprise, Version 7.1 (SAS Institute, Inc., Cary, NC, USA).

## Results

The study included 10 242 CCS and 49 068 matched general population controls (Table 1), who underwent 8911 and 34 750 COVID-19 tests, respectively. The distributions of sex and age were similar between survivors and controls. Approximately three-quarters of CCS were >10 years since cancer diagnosis at the time of COVID-19 testing, with a median age of 25 years. Among survivors, 28.5% were diagnosed with leukemia and 3.0% had undergone ABMT.

**Table 1:** Characteristics of childhood cancer survivors and matched general population controls.

**Characteristic**	**No. (% [95% CI])**		***P* value**
	**Childhood cancer survivors (*n* = 10 242)**	**Matched general population controls (*n* = 49 068)**	
Sex			
Male	5,581 (54.5 [52.4–56.6])	26 766 (54.5 [53.6–55.5])	0.91
Female	4,661 (45.5 [43.7–47.3])	22 302 (45.5 [44.6–46.3])	
Current age, y			
Median (IQR)	26 (14)	26 (14)	
5–9	420 (4.1 [3.7–4.5])	2,026 (4.1 [3.9-4.3])	0.99
10–20	2,418 (23.6 [22.5–24.7])	11,552 (23.5 [23.0-24.0])	
21–30	3,605 (35.2 [33.8–36.6])	17,249 (35.2 [34.5-35.8])	
≥30	3,799 (37.1 [35.6–38.6])	18,241 (37.2 [36.5-37.9])	
Age at cancer diagnosis, y			
Median (IQR)	6 (10)	NA	NA
Time since cancer diagnosis, y			
Median (IQR)	17 (14)	NA	NA
5–10	2,064 (20.2 [19.2–21.1])	9912 (20.2 [19.8–20.7])^a^	NA
>10	8,178 (79.9 [76.0–83.7])	39 156 (79.8 [78.0–81.6])^a^	
Initial cancer diagnosis			
Leukemia	2,918 (28.5 [27.3–29.7])	13 941(28.4 [27.9–29.0])^a^	NA
Lymphoma	1,730 (16.9 [16.0–17.8])	8340 (17.0 [16.6–17.4])^a^	
CNS	2,155 (21.0 [20.0–22.0])	10 314 (21.0 [20.6–21.5])^a^	
Other	3,439 (33.6 [32.2–35.0])	16 473 (33.6 [32.9–34.2])^a^	
Allogenic bone marrow transplant			
Yes	311 (3.0 [2.7–3.4])	1496 (3.0 [2.9–3.2])^a^	NA
No	9,931 (97.0 [86.0–107.9])	47 572 (97.0 [92.0–101.9])^a^	

a *Values are the count and distribution of the general population controls matched to childhood cancer survivors who have given characteristics*.

Overall, a significantly greater proportion of CCS underwent at least one COVID-19 test than controls (35.9 vs. 32.0%, RR =1.27 (95% CI 1.21-1.34); Table 2). This was consistent across subsets of CCS evaluated (Table 2), except among CCS who had undergone ABMT (*P* > 0.01; Table 2).

**Table 2:** COVID-19 testing, positivity and infection rates among childhood cancer survivors and matched general population controls.

**Subgroup**	**COVID-19 Testing Rate** ^**d**^			**COVID-19 Test Positivity Rate** ^**e**^			**COVID-19 Infection Rate** ^**f**^		
	**No. (%)** ^**a**^		**RR (95%CI)^**b**^**	**No. (%)** ^**a**^		**RR (95% CI)^**b**^**	**No. (%)** ^**a**^		**RR (95% CI)^**b**^**
	**Childhood cancer survivors** ** (*n* = 10 242)**	**Matched population controls** ** (n = 49 068)**		**Childhood cancer survivors** ** (*n* = 3680)**	**Matched population controls** ** (*n* = 15 695)**		**Childhood cancer survivors** ** (*n* = 10 242)**	**Matched population controls** ** (*n* = 49 068)**	
Total	3680 (35.9)	15 695 (32.0)	1.27 (1.21–1.34)^c^	157 (4.3)	856 (5.5)	0.71 (0.61-0.82)^c^	157 (1.5)	856 (1.7)	0.81 (0.70–0.92)^c^
Current age, y									
5–9	166 (39.5)	622 (30.7)	1.64 (1.31–2.07)^c^	4 (2.4)	27 (4.3)	0.72 (0.35–1.48)	4 (1.0)	27 (1.3)	1.01 (0.49–2.08)
10–19	795 (32.9)	3171 (27.5)	1.44 (1.30–1.60)^c^	41 (5.2)	199 (6.3)	0.64 (0.48–0.86)^c^	41 (1.7)	199 (1.7)	0.82 (0.62–1.10)
20–29	1440 (39.9)	6137 (35.6)	1.30 (1.20–1.41)^c^	72 (5.0)	343 (5.6)	0.79 (0.64–0.97)	72 (2.0)	343 (2.0)	0.89 (0.73–1.10)
≥30	1279 (33.7)	5765 (31.6)	1.14 (1.05–1.24)^c^	40 (3.1)	287 (5.0)	0.63 (0.47–0.83)^c^	40 (1.1)	287 (1.6)	0.66 (0.50–0.87)^c^
Time since cancer diagnosis, y									
5–10	792 (38.4)	3002 (30.3)	1.65 (1.48–1.83)^c^	38 (4.8)	165 (5.5)	0.66 (0.49–0.87)^c^	38 (1.8)	165 (1.7)	0.90 (0.68–1.19)
>10	2888 (35.3)	12 693 (32.4)	1.20 (1.13–1.26)^c^	119 (4.1)	691 (5.4)	0.72 (0.61–0.84)^c^	119 (1.5)	691 (1.8)	0.78 (0.67–0.92)^c^
Initial cancer diagnosis									
Leukemia	1055(36.2)	4443 (31.9)	1.16 (1.11–1.22)^c^	44 (4.2)	226 (5.1)	0.76 (0.58–0.99)	44 (1.5)	226 (1.6)	0.86 (0.66–1.12)
Lymphoma	645 (37.3)	2700 (32.4)	1.17 (1.10–1.24)^c^	26 (4.0)	167 (6.2)	0.58 (0.42–0.82)^c^	26 (1.5)	167 (2.0)	0.68 (0.49–0.94)^c^
CNS	753 (34.9)	3245 (31.5)	1.13 (1.06–1.20)^c^	34 (4.5)	190 (5.9)	0.72 (0.52–0.98)	34 (1.6)	190 (1.8)	0.82 (0.61–1.11)
Other	1227(35.7)	5307 (32.2)	1.12 (1.07–1.17)^c^	53 (4.3)	273 (5.1)	0.74 (0.57–0.95)	53 (1.5)	273 (1.7)	0.82 (0.64–1.05)
Allogenic bone marrow transplant									
Yes	111 (35.7)	494 (33.0)	1.19 (0.90–1.58)	6 (5.4)	19 (3.9)	1.22 (0.58–2.55)	6 (1.9)	19 (1.3)	1.36 (0.67–2.78)
No	3569 (35.9)	15 201 (32.0)	1.28 (1.21–1.34)^c^	151 (4.2)	837 (5.5)	0.69 (0.60–0.80)^c^	151 (1.5)	837 (1.8)	0.79 (0.69–0.91)^c^

a *Number and percentages are based on the number of individuals*.

b *From multivariable generalized estimating equations that include (where applicable) multiple tests per individual, for a total of 8911 COVD-19 tests among childhood cancer survivors and 34 750 COVID-19 tests among matched general population controls*.

c *Indicates that P < 0.01*.

d *COVID-19 testing rate is defined as the proportion of individuals undergoing COVID-19 testing at least once*.

e *COVID-19 test positivity rate is defined as the proportion of tested individuals with at least one positive COVID-19 test*.

f *COVID-19 infection rate is defined as the proportion of all subjects with at least one positive test*.

Among those tested, the overall likelihood of at least one positive test among CCS was significantly lower than matched controls (4.3 vs. 5.5%; RR = 0.71 (95% CI = 0.61–0.82); Table 2). This was true for most, but not all subgroups examined (Table 2).

Including all at-risk subjects, the overall risk of COVID-19 infection was lower among CCS than controls (1.5 vs. 1.7%; RR = 0.81 (95% CI = 0.70–0.92); Table 2) and, specifically among CCS ≥30 years, >10 years since diagnosis, previously diagnosed with lymphoma, and without prior ABMT (*P* < 0.01; Table 2).

## Discussion

To our knowledge, this is the largest study to evaluate the risk of COVID-19 infection among childhood cancer survivors, and the only one in which this risk is compared to matched general population controls. CCS did not appear to be at increased risk of COVID-19 infection, and on the contrary in some cases were less likely to be infected than matched controls. It may be that heightened health-related awareness (or anxiety) among CCS may both lower the threshold to seek out testing, and motivate more vigilant precautionary behavior that reduced overall COVID infection risk.

Although health care providers managing CCS care should be familiar with recommended precautions to reduce the risk of COVID-19 in this patient population (1), CCS-specific recommendations are, in fact, based on extremely limited data. A retrospective study of 321 CCS tested at one hospital in the United States reported that 10.9% (*n* = 35) of subjects had COVID-19-related symptoms, and 7.8% (n=20) tested positive for COVID-19 antibodies, which they noted was lower than the general population of the same age and location (4).

Prior evidence showed that long-term survivors of ABMT may be at greater risk of viral infection than the general population (5). However, we did not find that CCS of ABMT had significantly different rates of positive tests or infection compared to controls, but these results should be interpreted with caution due to the small sample size.

This study has limitations that warrant consideration. We did not have data regarding COVID-19 symptoms or exposure history and our findings cannot distinguish biologic susceptibility from behavioral contributors to infection risk. In addition, we did not have up-to-date data regarding hospitalization rates or other measures of COVID-19 severity, and could not evaluate whether infected survivors experience more severe symptoms than the general population. Finally, our results precede the widespread rollout of COVID-19 vaccination in Ontario, and so while we can be certain that the results do not reflect preferential vaccination of survivors, we cannot comment on the effectiveness of vaccination of CCS.

## Conclusions

The results provide reassurance that the risk of COVID-19 infection does not appear to be increased among CCS overall. As such, CCS and health care providers should adhere to CCS-specific recommendations to reduce COVID-19 infection risk. Future studies to examine whether CCS are more likely to experience severe COVID-19 related symptoms and morbidity would be valuable, as would an evaluation of the effectiveness of COVID vaccination among survivors, some of whom may have immune deficiencies related to prior treatment.

## Data Availability Statement

The data analyzed in this study is subject to the following licenses/restrictions: Data used in the analysis may be made available to investigators in a manner compliant with section 45 of Ontario's Personal Health Information Act. Requests to access these datasets should be directed to David.hodgson@rmp.uhn.ca.

## Ethics Statement

This study was conducted in accordance with the privacy policies governing the Pediatric Oncology Group of Ontario and the Institute for Clinical Evaluative Sciences and in compliance with section 45 of Ontario's Personal Health Information Protection Act.

## Author Contributions

MA had full access to all of the data in the study and take responsibility for the integrity of the data and the accuracy of the data analysis. DH and MA: concept and design. DH, MA, RM, FL, NB, and PG: acquisition, analysis, or interpretation of data. MA, FL, and DH: drafting of the manuscript. DH, FL, MA, RM, NB, and PG: critical revision of the manuscript for important intellectual content. MA and RM: statistical analysis. All authors contributed to the article and approved the submitted version.

## Funding

This work was funded the Ontario Ministry of Health. DH is supported by the Pediatric Oncology Group of Ontario Chair in Childhood Cancer Control, University of Toronto. The funders had no direct role in the analysis or the content of the manuscript.

## Conflict of Interest

The authors declare that the research was conducted in the absence of any commercial or financial relationships that could be construed as a potential conflict of interest.

## Publisher's Note

All claims expressed in this article are solely those of the authors and do not necessarily represent those of their affiliated organizations, or those of the publisher, the editors and the reviewers. Any product that may be evaluated in this article, or claim that may be made by its manufacturer, is not guaranteed or endorsed by the publisher.

## References

[1] Verbruggen LC Wang Y Armenian SH Ehrhardt MJ van der Pal HJH van Dalenet EC. Guidance regarding COVID-19 for survivors of childhood, adolescent, and young adult cancer: A statement from the International Late Effects of Childhood Cancer Guideline Harmonization Group. Pediatr Blood Cancer. (2020) 67:e28702. 10.1002/pbc.28702 32969160 PMC7537044

[2] Greenberg ML Barnett H Williams J. Atlas of Childhood Cancer in Ontario, 1985-2004. Toronto: Pediatric Oncology Group of Ontario (2015). Available online at: https://www.pogo.ca/research-data/data-reports/pogo-atlas/ (accessed May 26, 2021).

[3] Gupta S Pole JD. The validity of pediatric cancer diagnoses in a population-based general cancer registry in Ontario, Canada. BMC Cancer. (2016) 16:885. 10.1186/s12885-016-2931-8 27842503 PMC5109739

[4] Jimenez-Kurlander L Antal Z DeRosa A Diotallevi D Pottenger E Wilson N. COVID-19 in pediatric survivors of childhood cancer and hematopoietic cell transplantation from a single center in New York City. Pediatr Blood Cancer. (2021) 68:e28857. 10.1002/pbc.28857 33355979 PMC7883208

[5] Foord AM Cushing-Haugen KL Boeckh MJ Carpenter PA Flowers MED Leeet SJ. al. Late infectious complications in hematopoietic cell transplantation survivors: a population-based study. Blood Adv. (2020) 4:1232–41. 10.1182/bloodadvances.2020001470 32227211 PMC7160274

